# Particle attached and free floating pathogens survival kinetics under typical stream and thermal spring temperature conditions

**DOI:** 10.1186/s13568-018-0626-z

**Published:** 2018-06-19

**Authors:** Yi Wang, Pramod Pandey, Yawen Zheng, Edward Robert Atwill, Gregory Pasternack

**Affiliations:** 1 Department of Biological and Agricultural Engineering, University of California-Davis, Davis, CA 95616 USA; 2 Department of Population Health and Reproduction, School of Veterinary Medicine, University of California-Davis, 1089 Veterinary Medicine Drive, Davis, CA 95616 USA; 3 Department of Land, Air and Water Resources, University of California-Davis, Davis, CA 95616 USA

**Keywords:** Free floating pathogens, Particle attached pathogens, Survival in stream water and thermal spring conditions, Kinetics, *E. coli O157:H7 *, *Salmonella*, Public health, Sediment

## Abstract

Improved understanding of pathogen survival in the stream environment is needed to enhance existing predictive models of stream pathogen populations. Further, the increasing use of thermal springs for bathing necessitates additional studies focused on not only typical streams but also thermal spring conditions, where water temperature is relatively higher than typical streams. This study was conducted to assess the survival of *E. coli O157*:*H7* and *Salmonella* Typhimurium in stream water under free floating and particle-attached conditions at a range of temperature. A series of microcosm studies were conducted to determine pathogen decay rates. In bench-scale experiments, water circulation and sediment resuspension mimicked natural stream and thermal spring conditions, with continuous air flow providing aeration, constant mixing and turbulent conditions, and improved water circulation. Data on *E. coli O157:H7* and *Salmonella* survival were subsequently used to determine first-order decay equations for calculating the rate constant and decimal reduction time for the modeled experimental conditions. Results showed that at 40 °C, the survival of particle attached *E. coli* *O157:H7* was longer than that of particle attached *Salmonella*. Under free floating condition, *Salmonella* survived longer than *E. coli* *O157:H7*. At 50 °C, survival of particle attached *E. coli O157:H7* and *Salmonella* was longer than that of free floating *E. coli* and *Salmonella*. At 60 °C, survival of particle attached *Salmonella* was longer than that of free floating *Salmonella*. Similarly at 60 °C, the survival of *E. coli O157:H7  *under particle attached condition was longer than that of the free floating condition. The findings of this study suggest that the survival of *E. coli O157*:*H7* differs than the survival of *Salmonella* in stream water and thermal spring conditions, and the assumption used in previous studies to estimate survival of bacteria in stream environment could result in over/underestimation if the impact of particle attachment on pathogen survival is not accounted for.

## Introduction

The abundant pathogenic bacteria in ambient water bodies such as rivers, lakes, reservoirs, coasts and estuaries is a serious worldwide concern. As a form of pollution, pathogenic bacteria pose risks to human and animal health (Abia et al. 2016; Eichmiller et al. 2014; Pandey et al. 2016; Payment et al. 2000). In streams, these bacteria are reported to be in free floating condition, as well as in particle attached condition (Burton et al. 1987; Eichmiller et al. 2014; Munro et al. 2016; Pandey and Soupir 2013). Attachment to sediment particles changes bacteria survival and transport (Pandey et al. 2012).

While previous research studied the prevalence of pathogens in typical streams, understanding of waterborne and particle attached pathogen survival under thermal spring conditions (temp > 40 °C) is limited. Recent studies reported the prevalence of pathogenic organisms in geothermal springs (Di Filippo et al. 2017; Javanmard et al. 2017; Devi and Kanwar 2017). In many states of the US, the use of thermal springs for bathing is popular, particularly in Oregon and California, where these springs are found in mountains and deserts. Prevalence of pathogens (i.e., waterborne and particle attached) in these springs has a potential to pose health risks to users.

Under typical stream conditions, previous studies showed that streambed sediment acts as a reservoir of bacteria (Mueller-Spitz et al. 2010; Pachepsky and Shelton 2011; Pachepsky et al. 2017; Alm et al. 2003). The numbers of bacteria in streambed sediment can be considerably higher than those in the water column. Several fold higher concentrations of *E. coli* and *Cryptosporidium* oocyst are reported to persist in bed sediment compared to the water column (Bai and Lung 2005; Karim et al. 2004; Pandey et al. 2012). Subsequently, particle attached bacteria in streambed sediment are released by resuspension, which enhances bacteria concentrations in the water column (Cho et al. 2010; Pandey et al. 2012, 2016; Park et al. 2017). In this way, the streambed acts as a reservoir of pollution that can degrade water quality.

Further, streambed bacteria concentrations change water column quality considerably as flow and resuspension change (Bai and Lung 2005; Pachepsky and Shelton 2011; Pachepsky et al. 2017; Pandey and Soupir 2013; Pandey et al. 2012). While the role of resuspension in enhancing the bacterial pollution in stream water column is well reported (Cho et al. 2010; Kim et al. 2010; Pandey et al. 2012, 2016), how resuspended sediment particles influence the inactivation of pathogens, such as *E. coli* O157:H7 and *Salmonella* in the water column, is still not well understood.

One major challenge in calculating the growth and decay of pathogens in ambient water is that the inactivation rates of bacteria are system specific i.e., changes with water/environment conditions (Noble et al. 2004). As an example, the survival of bacteria, such as *Enterococcus,* is suggested to be longer than *E. coli* in freshwater, and the decay rates of *Salmonella* were higher than *Enterococcus* in coastal water (Craig et al. 2003; Haller et al. 2009). Compared to *Salmonella*, the survival of *E. coli* was found to be higher in estuaries (Chandran and Hatha 2005). While comparing survival of indicator microorganisms in aquatic systems, studies also showed that the survival of bacteria was not dependent upon the original source of contamination. For instance, the results of an inactivation study of indicator micro-organisms sourced from sewage influent, sewage effluent, and urban storm drain run-off, showed that the survival of indicator bacteria was similar in fresh and sea water. The degradation rate of *Enterococcus* was higher than *E. coli* in both fresh and seawater (Noble et al. 2004).

The survival of pathogens is likely to change when these pathogens are attached to sediment and clay particles present in natural water. Results of a comparative survival study in water column and sediment showed that the decay of *Salmonella* was reduced in coastal water sediment (Craig et al. 2003) compared to the decay in the water column indicating that sediment particles act as a protective factor for bacterial survival. In general, sediment enhances growth and reduces decay of fecal indicator bacteria and other organisms potentially due to nutrient rich environment (Haller et al. 2009). However, the impacts of sediment on survival of pathogens can be species specific. Results of the survival of *E. coli*, *Salmonella*, *Vibrio cholerae* and *Shigella dysenteriae* in riverbed sediments showed that only *E. coli* and *Salmonella* survived at low temperature (4 °C) for 28 days (Abia et al. 2016). The survival rate of *V. cholera* was the shortest. However, at a significantly higher temperature (30 °C) the survival of *Salmonella* and *V. cholera* was greater than *E. coli* and *S. dysenteriae*, which indicates that the survival of particle attached pathogens changes with temperature. While these previous results provide important insights with regards to bacteria survival in typical stream conditions, where the temperature is less than 30 °C, the understanding of microbial pathogen survival in thermal spring conditions is rare. Many of these thermal springs possess the water temperature in a range of 50–60 °C. It is reported that the survival curves of bacterial spores is changed at higher temperature, which affects inactivation time and process (Cerf 1977). The increased uncertainty in bacterial inactivation is reported as a result of combined effect of sediment particle size and temperature (Howell et al. 1996). The presence of sediment particles and warm conditions resulted in extended survival of fecal coliform. Further, increased half-lives and regrowth of fecal coliform were observed, when fecal coliform was enumerated in feces-amended sediment under various temperature conditions (Howell et al. 1996).

In addition to temperature, many factors such as sunlight, dissolved oxygen, grazing and pH are known to influence pathogen survival (Brookes et al. 2004; Hipsey et al. 2008; Kohn and Nelson 2007; Schultz-Fademrecht et al. 2008; Sinton et al. 2007). In order to get protection against these detrimental factors in a natural environment, microorganisms colonize the surface of particles, such as sediment, phytoplankton, and zooplankton (Dang et al. 2008; Dang and Lovell 2000, 2002, 2016). The attachment and surface association with the particles are advantageous to the microbes in terms of protection from predators, viruses, solar radiation, chemicals, and other environmental factors that increase their mortality (Dang and Lovell 2016). In general, temperature is considered to be a dominant factor affecting growth and survival of microbes. Temperature-dependent growth and decay models are often used to predict bacteria/pathogen concentrations in streams (Alvarez et al. 2000; Pandey et al. 2012, 2016; Park et al. 2017; Spinks et al. 2006). Previous observations of instream *E. coli* numbers showed that *E. coli* concentrations in streams change with ambient temperature (Kim et al. 2010; Pandey et al. 2016). Furthermore, the temperature range from 55 to 65 °C is effective for eliminating enteric/pathogenic bacteria (Spinks et al. 2006). Lab-based microcosm studies that test the effect of temperature on pathogenic bacteria and virus survival also showed that increase in temperature results in reduced pathogen concentrations (Abia et al. 2016; Popat et al. 2010; Spinks et al. 2006).

The results of a thermal inactivation study, conducted for assessing water-borne pathogenic and indicator bacteria survival, showed that the strains of *Enterococcus faecalis*, *Shigella sonnei*, *E. coli* O157:H7 were more heat resistance than non-pathogenic *E. coli* O3:H6, *Salmonella*, and *Pseudomonas aeruginosa* (Spinks et al. 2006). Another microcosm study showed that the survival of *E. coli* and *Salmonella* in sewage treated by stabilization ponds was similar, while in brackish water the survival rate of *Salmonella* was greater than that of *E. coli* (Mezrioui et al. 1995).

While numerous previous studies provide crucial information in terms of pathogen survival in various water types and environmental conditions, additional insights are needed to understand the effect of a range of temperature (i.e., from mesophilic to thermophilic) on the survival of pathogens in streams and thermal spring conditions (Abia et al. 2016; Hipsey et al. 2008; Schultz-Fademrecht et al. 2008). While calculating pathogen survival in watershed scale models, often the decay rate for free floating and particle attached microorganisms is estimated using a single equation, which has a potential to produce erroneous results. Decay of particle attached pathogens is yet to be known for the stream environment, as well as for thermal spring conditions. Thus, the three objectives of this study involved assessing pathogen survival in (1) mesophilic (typical stream) and (2) thermophilic temperature conditions (thermal spring), considering free floating and particle attached conditions in both cases and (3) calculating and comparing the survival kinetics of two species under free floating and particle attached conditions. For all three objectives, *E. coli* O157:H7 and *Salmonella* are the test species. This study advances the basic science of pathogen ecology by reducing the uncertainty in pathogen survival and decay rates. By enhancing predictive modeling of stream pathogen populations this study facilitates better water quality management.

## Materials and methods

**Fig. 1:**
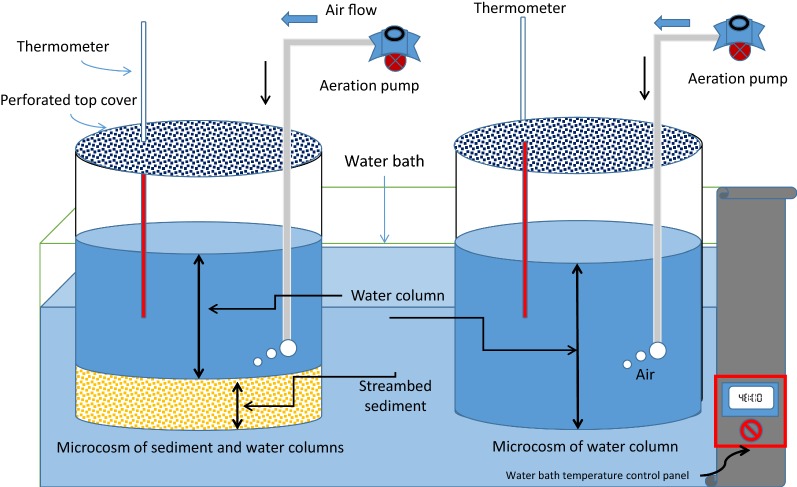
Experiment sketch of microcosm study (left shows the microcosm of sediment and water column, and right shows water column microcosm)

To understand *E. coli* and *Salmonella* survival in streams, a series of experiments were executed at multiple temperatures (Fig. 1) representing typical stream and thermal spring water temperature conditions. Experiments were performed in microcosms containing streambed sediment and stream water. The sediment was collected from the streambed. Water was collected from overlying water in streams. The sediment and water samples were collected from 10 randomly selected locations in four sub-watersheds of Merced River in Central Valley of California, USA. These sub-watersheds include (from downstream to upstream): Ingalsbe Slough-Merced River Watershed, Maxwell Creek-Merced River Watershed, Bear Creek-Merced River Watershed, and Yosemite Creek-Merced River Watershed. Sediment samples (approximately 20 g from each location) were mixed to create a composite sediment bed (≈ 3 cm depth; ≈ 200 g weight) in microcosms for inactivation study. Similarly, water samples (100 mL from each location) from 10 different locations were mixed to create a composite stream water column (≈ 10 cm depth) in microcosms.

Two microcosms were designed: (1) a microcosm with water and sediment (MWS); and (2) a microcosm with water only (MW). Both microcosms were used to study *E. coli* *O157:H7 * and *Salmonella* inactivation, but differed in that they addressed particle attached or free floating conditions, respectively. Aquarium pumps (Tetra, Whishper Air Pump, Model Number 77,846, size 10 gallon) were used for injecting air into a microcosm to maintain continuous water circulation and provide oxygen. Thermometers (Fisher Scientific) were placed in each microcosm to observe water column temperature. Both microcosms were placed in a water bath (Precision Scientific Thelco Water Bath Model 82), and whole temperature was regulated as needed. Temperature was monitored continuously to record the time required for reaching the desired temperature. Mesophilic experiments had water bath temperatures of 30 and 40 °C (a representative temperature of typical streams), while thermophilic experiments had water bath temperatures of 50 and 60 °C (a representative temperature for thermal spring conditions). Each experiment was performed twice. Run 1 indicates the first experiment and Run 2 indicates the second experiment for each temperature and organism. To develop the decay curves, the average of linear curves of Run 1 and Run 2 were used. One-Way ANOVA was implemented to test the significant differences among regression lines, *k*-values, and *z*-values (at *p* < 0.05).

**Fig. 2:**
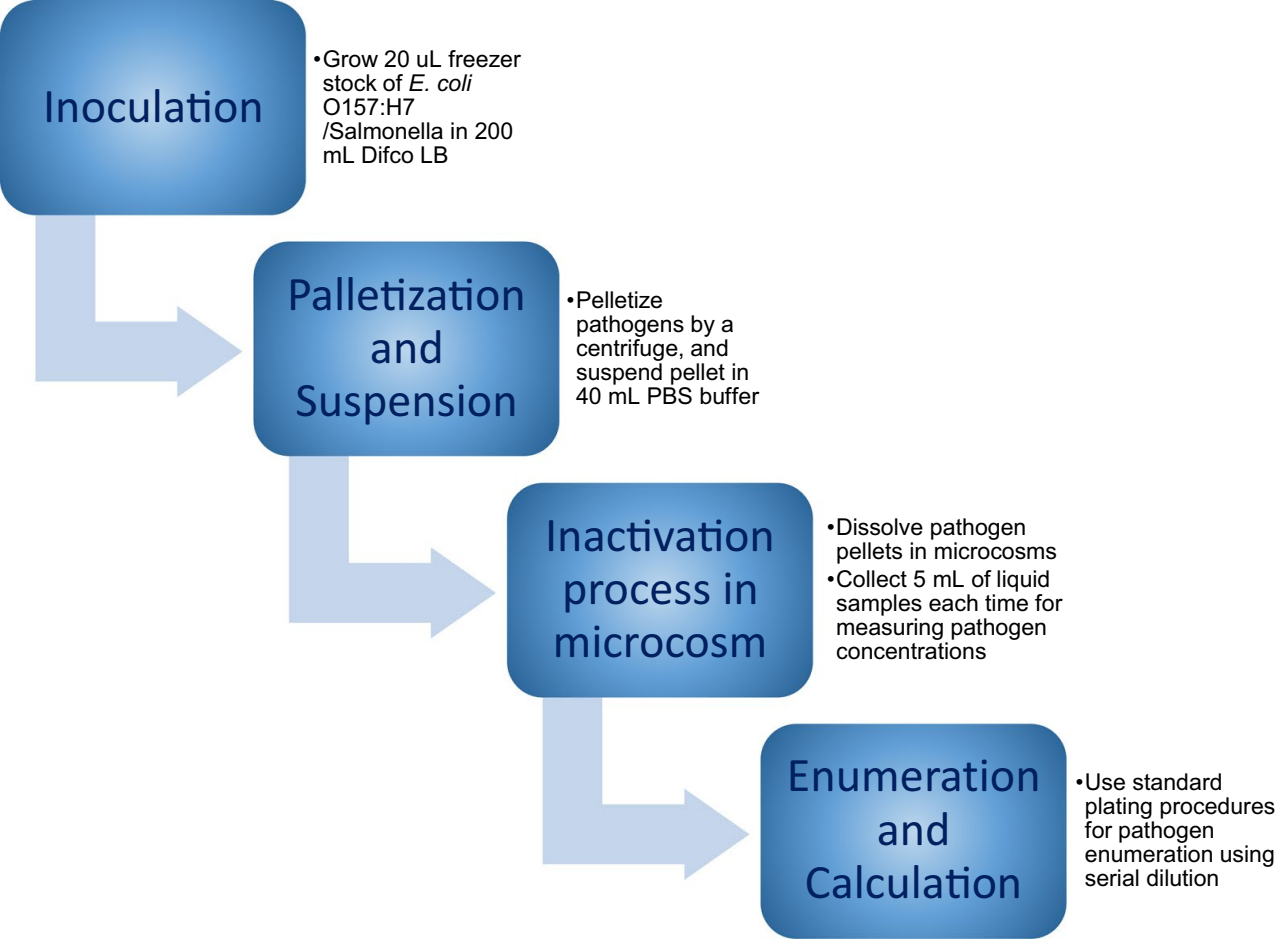
Steps involved in inactivation study of *E. coli O157:H7 * and *Salmonella* in microcosms

The steps involved in culture preparation and inoculations are shown in Fig. 2. To inoculate the feedstock, 20 μL freezer stocks of *E. coli* *O157:H7 * (ATCC #35150) and *Salmonella* Typhimurium LT2 (ATCC #700720) were grown in Difco LB (Luria–Bertani) Broth Miller growth media for 24 h at 37 °C under shaking conditions (200 rpm) (ThermoFisher Sci., MaxQ 4000). The culture was pelletized using a centrifuge (ThermoFisher Sci., Sorvall Legend X1R) at 5000 rpm for 20 min. Subsequently, the pellet was suspended in a 40-mL Phosphate Buffer Solution (PBS) buffer. This buffer solution with pathogens was dissolved in microcosms. After inoculation, gentle mixing was provided to both microcosms (MWS and MW) to distribute pathogens uniformly inside the microcosms.

During a survival study, 5-mL liquid samples of overlying water from the MWS and MW were collected over time for *E. coli O157:H7 * and *Salmonella* cell enumeration. Samples were serially diluted in PBS buffer, and several levels of diluted samples were plated in selective agar plates. Each sample was analyzed in duplicate. MacConckey II agar with sorbitol (BBL, Becton, Dickinson and Company, Sparks, MD, USA), a selective and differential media for the detection of sorbitol-nonfermenting *E. coli*, was used for the enumeration of *E. coli* *O157:H7* colonies. Difco XLD Agar (Becton, Dickinson and Company, Sparks, MD, USA) was used for enumerating the colonies of *Salmonella*. After plating, MacConckey agar plates were incubated at 37 °C for 24 h following U.S. Food and Drug Administration’s bacteriological analytical manual procedure. Pathogen growth was enumerated using a colony counter. *E. coli* *O157:H7* produced colorless colonies, while *Salmonella* produced the colonies with red-yellow with black centers. Finally, pathogen number in per unit mass (mL) of original was calculated based on colony numbers obtained from each sample.

The observations of *E. coli O157:H7 * and *Salmonella* were used to derive the first order decay equations to determine the relationships between time and survival of pathogens. The changes in *E. coli O157:H7 * and *Salmonella* numbers were fitted into logarithmic lines [i.e., Log (CFU/mL) versus time (min)] at a temperature. Logarithmic reduction of pathogens was used to estimate decimal reduction time (*D*-value), which indicates the required heating time in minutes at a temperature for reducing pathogens by a factor of 10. The *D*-value was estimated as the negative of the inverse of slope of the linear line between pathogen numbers [log (CFU/mL)] and time (min) (Eq. 1).

1 $$D = - 1/S$$

where *S* is a slope of a linear line between *E. coli O157:H7 * (or *Salmonella*) numbers [log (CFU/mL)] and time (min).

The first order rate constant (*k*) in min^−1^ was estimated from the *D*-value.

2 $$k = 2.303/D$$

*Z*-value, which determines the number of degrees of temperature change required for changing the *D*-value by a factor 10, was derived from Arrhenius equation (Eq. 3)

3 $$Z = - \left( {\frac{{T_{1} - T_{2} }}{\log D1 - \log D2}} \right)$$

*D*_*2*_ and *D*_*1*_ are D-values (min) corresponding to temperature *T*_*2*_ and *T*_*1*_ (°C).

## Results

### Temperature and microcosm-type responses

**Fig. 3:**
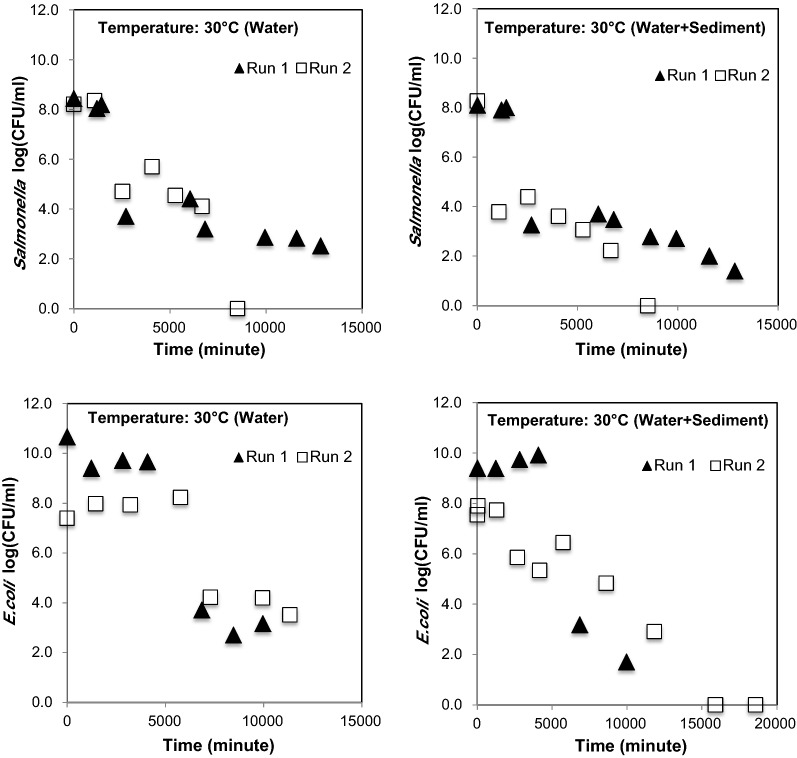
Survival of *Salmonella* and *E. coli O157:H7 * and at 30 °C (mesophilic temperature) in free floating and particle attached conditions

**Fig. 4:**
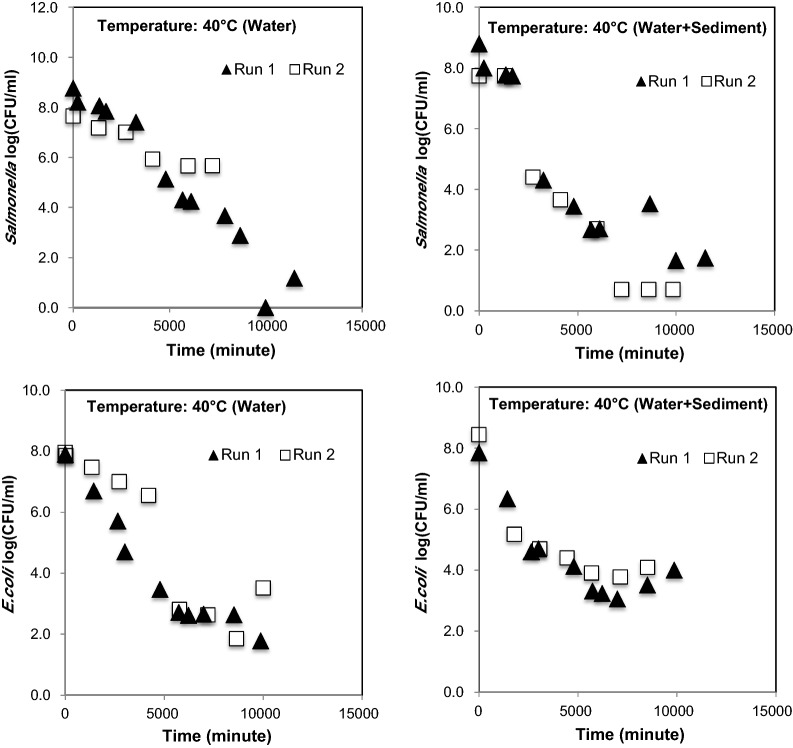
Survival of *Salmonella* and *E. coli O157:H7 * and at 40 °C (mesophilic temperature) in free floating and particle attached conditions

The initial temperature of stream water was ≈ 15 °C. The time required for reaching desired temperature for 60 and 50 °C was comparable. However, the time required for reaching the desired temperature in the microcosms with sediment was slightly delayed compared to the time in the microcosm with no sediment. In general, approximately 25–30 min was needed to reach the desired temperature of stream water in microcosms. *E. coli O157:H7 * and *Salmonella* survival (Fig. 3) was comparable at 30 °C. At 40 °C, survival of particle attached *E. coli O157:H7 * was longer than free floating condition. The survival of *Salmonella* was shorter than *E. coli* *O157:H7 * under particle attached condition, while under free floating condition, *Salmonella* survived longer than *E. coli* *O157:H7 * (Figs. 3, 4). At 30 °C, *Salmonella* numbers reduced from 8 orders of magnitude to 2 orders of magnitude in 12,840 min in Run 1, and changed from 8 orders of magnitude to non-detectable in Run 2 in 8516 min. At 40 °C, *Salmonella* numbers (under free floating conditions) changed from 8 orders of magnitude to 5 orders of magnitude in 11,490 min in Run 1, while in Run 2 it changed from 7 orders of magnitude to 5 orders of magnitude in 7232 min. Under particle attached conditions, *Salmonella* numbers changed from 8 orders of magnitude to 1 order of magnitude in 11,490 min and from 7 orders of magnitude to less than 1 order of magnitude in 9854 min.

**Fig. 5:**
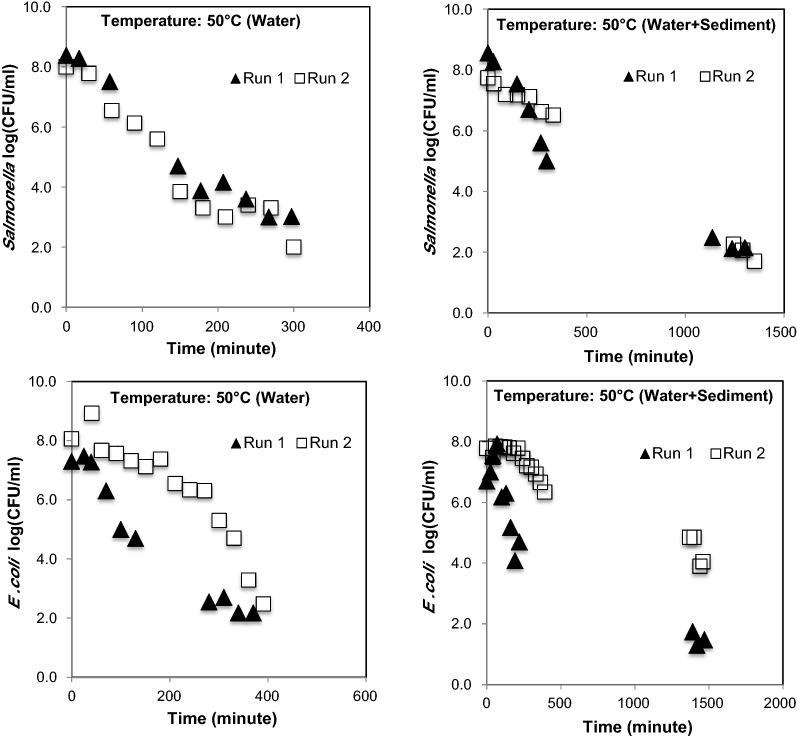
Survival of *Salmonella* and *E. coli* *O157:H7 * and at 50 °C (thermophilic temperature) in free floating and particle attached conditions

At 50 °C, the *E. coli O157:H7 * and *Salmonella* survival under particle attached condition was longer than free floating condition (Fig. 5). The *E. coli O157:H7 * under free floating condition at 50 °C (Run 1) changed from 7 orders of magnitude to 2 orders of magnitude in 370 min. In Run 2, *E. coli O157:H7 * changed from 8 orders to 2 orders in 391 min. Under particle attached condition, *E. coli* *O157:H7 * changed from 6 to 1 order of magnitude in 1470 min in Run 1, and in Run 2, it changed from 7 orders of magnitude to 4 orders of magnitude in 1461 min.

At 50 °C, *Salmonella* was reduced from 8 orders of magnitude to 3 orders of magnitude (under free floating condition) in 297 min in Run 1, and in Run 2, it changed from 8 orders of magnitude to 2 orders of magnitude in 300 min (Fig. 5). Under particle attached condition, *Salmonella* was reduced from 8 orders of magnitude to 2 orders of magnitude in 1302 min and from 7 orders of magnitude to one order of magnitude in 1350 min.

**Fig. 6:**
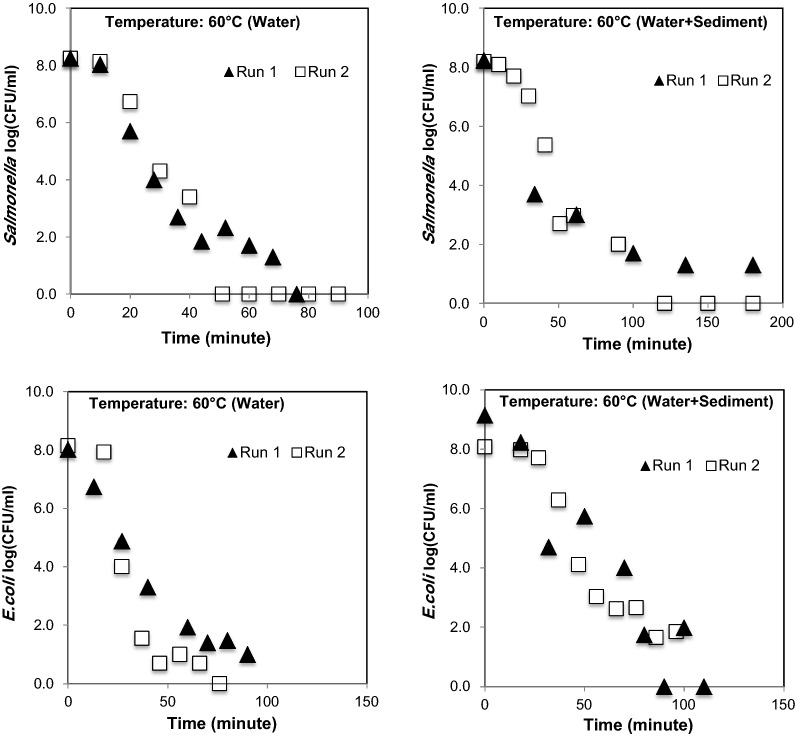
Survival of *Salmonella* and *E. coli O157:H7 * and at 60 °C (thermophilic temperature) in free floating and particle attached conditions

At 60 °C, the *Salmonella* was reduced from 8 orders of magnitude to non-detectable numbers in less than 76 min under free floating conditions, and in less than 121 min under particle attached condition. *E. coli O157:H7 * under free floating condition was reduced from 8 orders of magnitude to non-detectable numbers in less than 90 min, and under particle attached condition, *E. coli O157:H7 * survived longer than 100 min at 60 °C (Fig. 6).

While comparing the results of pathogen survival at mesophilic temperature (30 °C and 40 °C) with high temperature (50 °C and 60 °C), certain expected differences in the colony number (CFU/mL) were observed among Run 1 and Run 2 (Figs. 3, 4, 5) data. These differences among two experiments could have been due to the impact of air injection, temperature, and combined effects of temperature, air, and particle, which were beyond control. Therefore, the average of two datasets (obtained from two independent experiments i.e., Run 1 and Run 2) were used for determining the decay curves. The interferences in bacterial survival, half-life, and mortality rate due to combined effect of temperature and particles are well reported elsewhere, when the experiments were performed under multiple temperature conditions (Howell et al. 1996; Cerf 1977).

### Decay curves

**Fig. 7:**
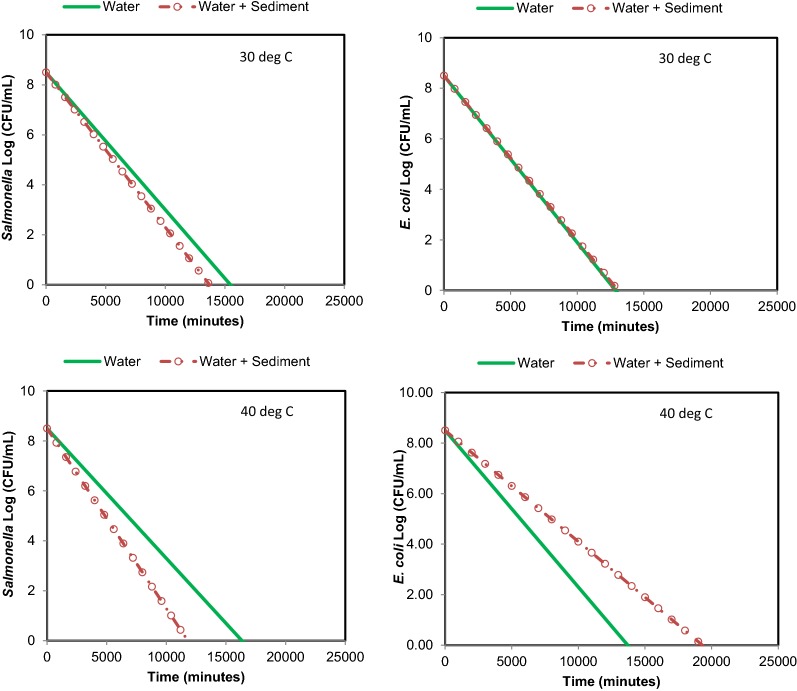
Decay curves of *E. coli O157:H7 * and *Salmonella* at 30 and 40 °C under free floating and particle attached conditions (curves shown are the average of curve for Run 1 and curve for Run 2; equations are shown in Table 1)

**Fig. 8:**
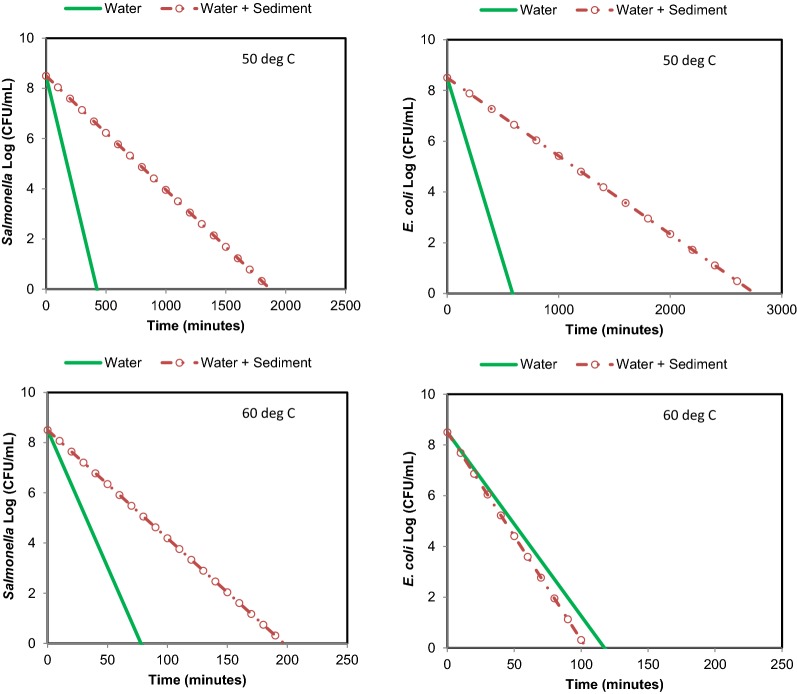
Decay curve of *E. coli* *O157:H7 * and *Salmonella* at 50 and 60 °C under free floating and particle attached conditions (curves shown are the average of curve for Run 1 and curve for Run 2; equations are shown in Table 1)

Linear fits on observations were carried out to determine the first-order decay equations for establishing the relationships between time and pathogen reduction at a temperature. The linear fits between time (min) and pathogen numbers (log CFU/mL) resulted in *R*^2^ between 0.75 and 0.99. Table 1 shows the equations for *E. coli* *O157:H7 * and *Salmonella* reductions for free floating and particle attached conditions at mesophilic and thermophilic temperatures. The decay curves for *E. coli O157:H7 * and *Salmonella*, using equations (Table 1), under mesophilic (stream) and thermophilic (spring) conditions are shown in Figs. 7, 8. The curves indicate that the survival of *E. coli O157:H7 * under free floating condition and particle attached condition was identical at 30 °C (typical stream). Similarly, *Salmonella* survival under free and particle attached condition was comparable at this temperature.

**Table 1:** Kinetics of *E. coli O157:H7* and *Salmonella* survival in free-floating and particle-attached conditions in stream water (time is in minute)

Temp. (°C)	*E. coli O157:H7*		*Salmonella*	
	Free-floating	Particle attached	Free-floating	Particle attached
	Equations of decay curves^2^			
30	^a^y = − 0.00066 × (time) + C_0_	^a^y = − 0.00065 × (time) + C0	^a^y = − 0.00055 × (time) + C0	^a^y = − 0.00062 × (time) + C0
40	^b^y = − 0.00062 × (time) + C0	^b^y = − 0.00044 × (time) + C0	^b^y = − 0.00052 × (time) + C0	^b^y = − 0.00072 × (time) + C0
50	^c^y = − 0.01448 × (time) + C0	^d^y = − 0.00308 × (time) + C0	^e^y = − 0.02000 × (time) + C0	^f^y = − 0.00454 × (time) + C0
60	^g^y = − 0.09910 × (time) + C0	^h^y = − 0.08188 × (time) + C0	^i^y = − 0.10908 × (time) + C0	^j^y = − 0.04308 × (time) + C0
	First order rate constant [*k*]^3^ (min^−1^)			
^a^30	0.00152	0.00150	0.00128	0.00143
^b^40	0.00143	0.00092	0.00120	0.00165
^c^50	0.03334	0.00709	0.04606	0.01044
^d^60	0.22822	0.18857	0.25120	0.09920
	Z-value^4^			
	9.14746	8.65426	8.30772	11.32888

Different letters in subscript of regression equations shows the line is significantly different at *p* < 0.05

Similar letters in subscript of *z*-*values* show the values are not significantly different at *p* < 0.05

Different letters in subscript of *k*-*values* show that these values were significantly different among temperature at *p *< 0.05

^1^C_0_ = initial pathogen concentration (CFU/mL)

^2^R^2^ values for decay curve varied between 0.75 and 0.99

^3^Rate constant (k) was estimated from D-values (k = 2.303/D)

^4^The Z-values were estimated based on slope (i.e., z-value = − 1/(slope between temperature and D-value)

**Fig. 9:**
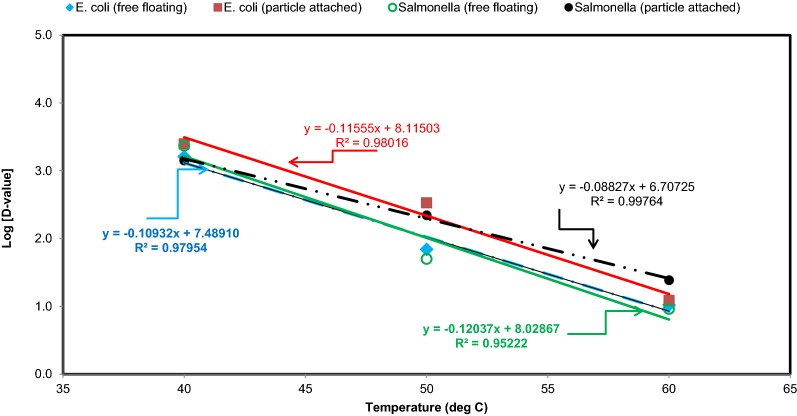
Relationships between temperature and thermal reduction time (D-value) for *Salmonella* and *E. coli O157:H7 *. The D-values were estimated from Table 1 [D-value = − 1/(slope)]

Decimal reductions time (*D*-value) was estimated using the first-order decay curve equations. At low temperature (stream environment), D-value was lower than that of higher temperature (spring environment). In general, D-values (Fig. 9) for *Salmonella* were higher under particle attached conditions compared to free floating conditions. Similarly, D-values for *E. coli O157:H7 * under particle attached conditions were larger than that of free floating conditions. However, these changes were temperature dependent, indicating that the survival of pathogens likely to change under particle attached condition depending on the ambient temperature.

The reaction rate constant (*k*) in min was estimated using D-values (*k *=* 2.303*/*D*), which is shown in Table 1. The rate constants under particle attached condition were lower than free floating conditions for both *E. coli O157:H7 * and *Salmonella*. The *Z*-value (*z *=− *1*/*slope*), which determines the number of degrees Celsius required to change a *D*-value by one factor of 10, are also shown in Table 1. Under free floating condition, Z-values for *E. coli* *O157:H7 * and *Salmonella* were 9.1 and 8.2, respectively, while under particle attached condition, these values were 8.6 and 11.2.

Comparison of the results of *E. coli O157:H7 * and *Salmonella* survival in free floating and particle attached conditions indicates that the survival of pathogens was increased in the latter for the same temperature. At lower temperature (typical stream ≈ 30 °C), the difference among *D*-values of free floating and particle attached conditions was lower than that at higher temperature. At thermophilic temperature (thermal spring ≈ 40–50 °C), the *D*-value of particle attached *E. coli O157:H7 * condition was around 5 times of free floating *E. coli O157:H7 *. The D-value of *Salmonella* for particle attached condition was 4.3 times of that of free floating conditions.

## Discussion

The results of this study suggest that the time required for pathogen reduction at a temperature (either typical streams or thermal spring) could be higher when stream water has suspended particles, depending on the ambient temperature. These results align with previous research (Abia et al. 2016; Bai and Lung 2005; Burton et al. 1987; Havelaar et al. 1995; Mueller-Spitz et al. 2010), which suggested that sediment particles provide favorable environments for the survival of bacteria such as *E. coli*, *Salmonella*, *V. cholerae* and *S. dysenteriae*.

The current study provided the observations needed for developing models capable of calculating pathogen survival under particle attached condition, which is not well established. The mechanism that could explain the study findings is that sediment particles provide improved nutrient availability and resistance to heat stress. As a result, the resuspension of sediment particles sustains individual pathogens and ultimately enhances the pathogen population in stream water (Craig et al. 2001; Droppo et al. 2011; Mueller-Spitz et al. 2010; Pandey and Soupir 2013; Pandey et al. 2016). Despite the common understanding of enhanced pathogen survival in sediment, there is a knowledge gap in terms of changes in pathogen survival and decay kinetics under particle attached conditions during resuspension (Brookes et al. 2004; Hipsey et al. 2008; Pachepsky and Shelton 2011), and the results of this study provide the much needed additional information to fill the knowledge gap. Further, considering the increased uses of thermal springs for bathing purposes, the scope of this study is wide.

In general, temperature-based decay curves are used for calculating in-stream pathogen survival, which is often augmented in hydrological models (Calero-Cáceres et al. 2017; Cho et al. 2010; Droppo et al. 2011; Pandey et al. 2016; Payment et al. 2000). Therefore, it is necessary to understand how the behavior of these equations changes with temperature and sediment resuspension conditions. Although previous studies evaluated the survival of pathogenic bacteria in various freshwater sediment (Burton et al. 1987; Calero-Cáceres et al. 2017; Munro et al. 2016; Eichmiller et al. 2014), and linear models are proposed to describe the die-off rates, only few if at all any study compared the survival of *E. coli O157:H7* and *Salmonella* in free floating and resuspended sediment conditions at typical stream and thermal spring conditions.

This study showed that the decay curve changes considerably with change in temperature. The differences in the decay rate constants among pathogens (such as *E. coli O157:H7* and *Salmonella*) were greater at high temperature (thermophilic temperature over mesophilic temperature). The impact of particle resuspension on *D*-value was evident; however, the effect was greater at relatively high temperature (> 40 °C ≈ thermal spring conditions). The free floating and particle attached pathogen survival study provides pathogen specific information useful for calculating the public and animal health risks caused by contaminated stream water. Future studies involving other organisms such as fecal indicator, *Cryptosporidium* spp., *Giardia* spp. *Shigella* spp., *Vibrio* spp. *Clostridium* spp., *Staphylococcus aureus* and multiple human enteric viruses to assess the impact of temperature and resuspension under multiple flow conditions will provide further needed information to improve existing understanding of pathogen survival in stream and thermal spring conditions. We anticipate that the results of this study will provide improved insights of pathogen survival in stream and spring water. The decay equations and observations provided here will be potentially useful for future hydrological modelling studies intended for calculating pathogen/pathogen indicator concentrations in ambient waterbodies such as typical streams and thermal springs.

## Abbreviations

CFU: colony forming units
D-value: decimal reduction time
MWS: microcosm with water and sediment
MW: microcosm with water only
